# Supplementary material for: Probabilities of developing HIV-1 bNAb sequence features in uninfected and chronically infected individuals
Source: Nat Commun. 2023 Nov 6;14:7137. doi: 10.1038/s41467-023-42906-y (PMC10628170; doi:10.1038/s41467-023-42906-y)
Supplement: Supplementary file 1 — Supplementary Information [file 41467_2023_42906_MOESM1_ESM.pdf]

Supplementary Information

"Probabilities of developing HIV-1 bNAb sequence features in uninfected and chronically infected individuals"

Supplementary Figures

Supplementary Figure 1	Gating strategy to isolate naive or antigen-experienced B cells
Supplementary Figure 2	Unique molecular identifier copy number distribution of fully annotated reads
Supplementary Figure 3	Spike-in experiment with different percentages of known cell line clones
Supplementary Figure 4	Influence of viral panel composition on the determination of neutralization breadth and potency
Supplementary Figure 5	Correlations of 70 bNAb heavy chain sequence features and probability values
Supplementary Figure 6	Light chain probabilities of broadly neutralizing antibodies
Supplementary Figure 7	Comparison of V gene segment insertions and deletions in IgG heavy chains between cohorts
Supplementary Figure 8	IgG heavy and light chain repertoire characteristics, stratified by antiretroviral treatment
Supplementary Figure 9	IgG heavy and light chain repertoire characteristics, stratified by neutralization activity

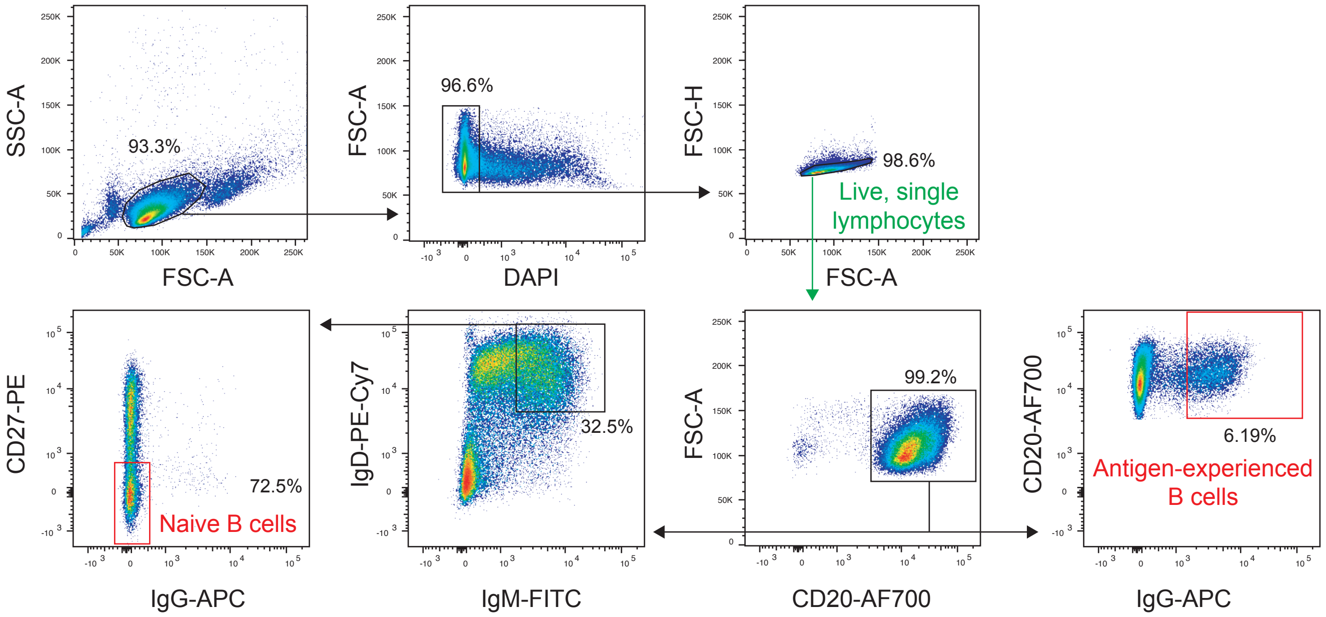

Supplementary Figure 1: Gating strategy to isolate naive or antigen-experienced B cells. Gating includes the pre-selection of the lymphocyte population, exclusion of dead cells and doublets, as well as selection of $CD20^+$ cells. From this population, antigen-experienced are defined as IgG^+ and naive B cells as IgD^+/IgM^+ and $CD27^-/IgG^-$. Scatter plots show a representative example from an uninfected control individual. Numbers indicate percentages of cells in the depicted gates. Gating on antigen-experienced IgG^+ B cells was applied to the samples depicted in Figs. 1b, 1f, and 3a. Gating on naive B cells was applied on samples in Supplementary Figure S3.

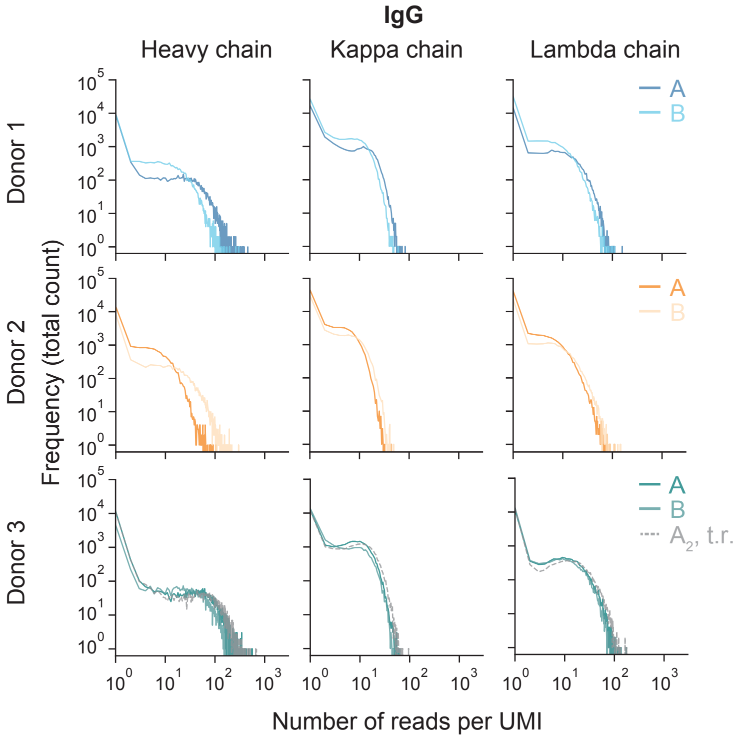

Supplementary Figure 2: Unique molecular identifier copy number distributions of fully annotated reads. Numbers of fully annotated reads per UMI were counted (x-axis) and the frequency of UMI groups with identical count numbers determined (y-axis). Colored solid lines represent biological replicates (*n*=2), dashed lines represent a technical replicate (t.r., donor 3, sample A_2). The technical replicate was processed independently from all other samples on a different day and sequenced in a separate NGS run. Source data are provided as a Source Data file.

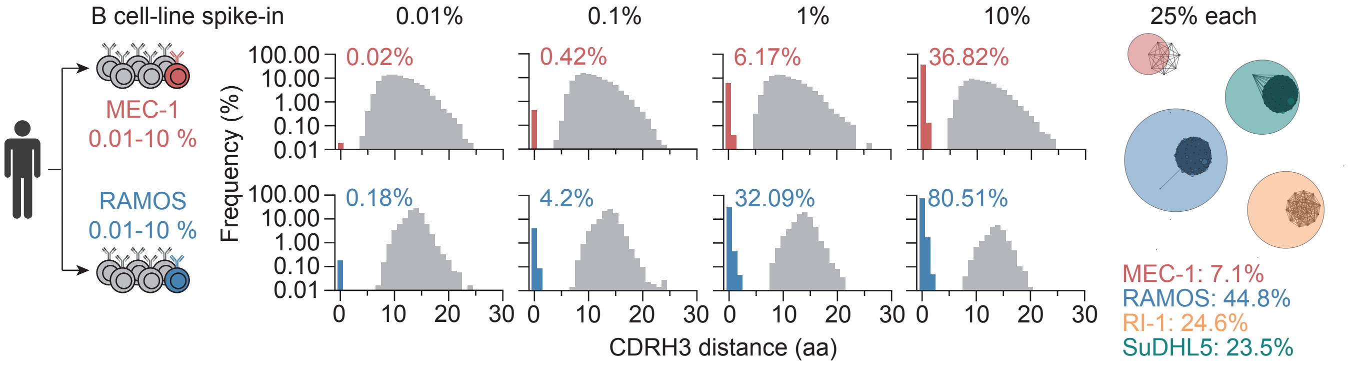

Supplementary Figure 3: Spike-in experiment with different percentages of known cell line clones. Naive IgM+ B cells from a blood donor were spiked with different concentrations (0.1 - 10%) of tumor B cell lines (MEC-1 and RAMOS) to analyze in total 100,000 cells. Plots show amino acid (aa) distances of all reconstituted CDRH3s from the individual spike-in experiments in comparison to the cell line CDRH3. Colored bars depict CDRH3s with <4 amino acids difference to the cell line CDRH3 and colored percentages represent their fraction of all comparisons. As an internal control, a sample comprising 25,000 cells from each of the four depicted cell lines was processed for repertoire sequencing. Each node represents a unique CDRH3. The node size is proportional to the frequency among all identified CDRH3s. Nodes are connected if they share at least 75% of their CDRH3 amino acid sequence. Nodes are colored according to the cell lines if they share at least 75% of the CDRH3 amino acid sequence with a cell line. Source data are provided as a Source Data file.

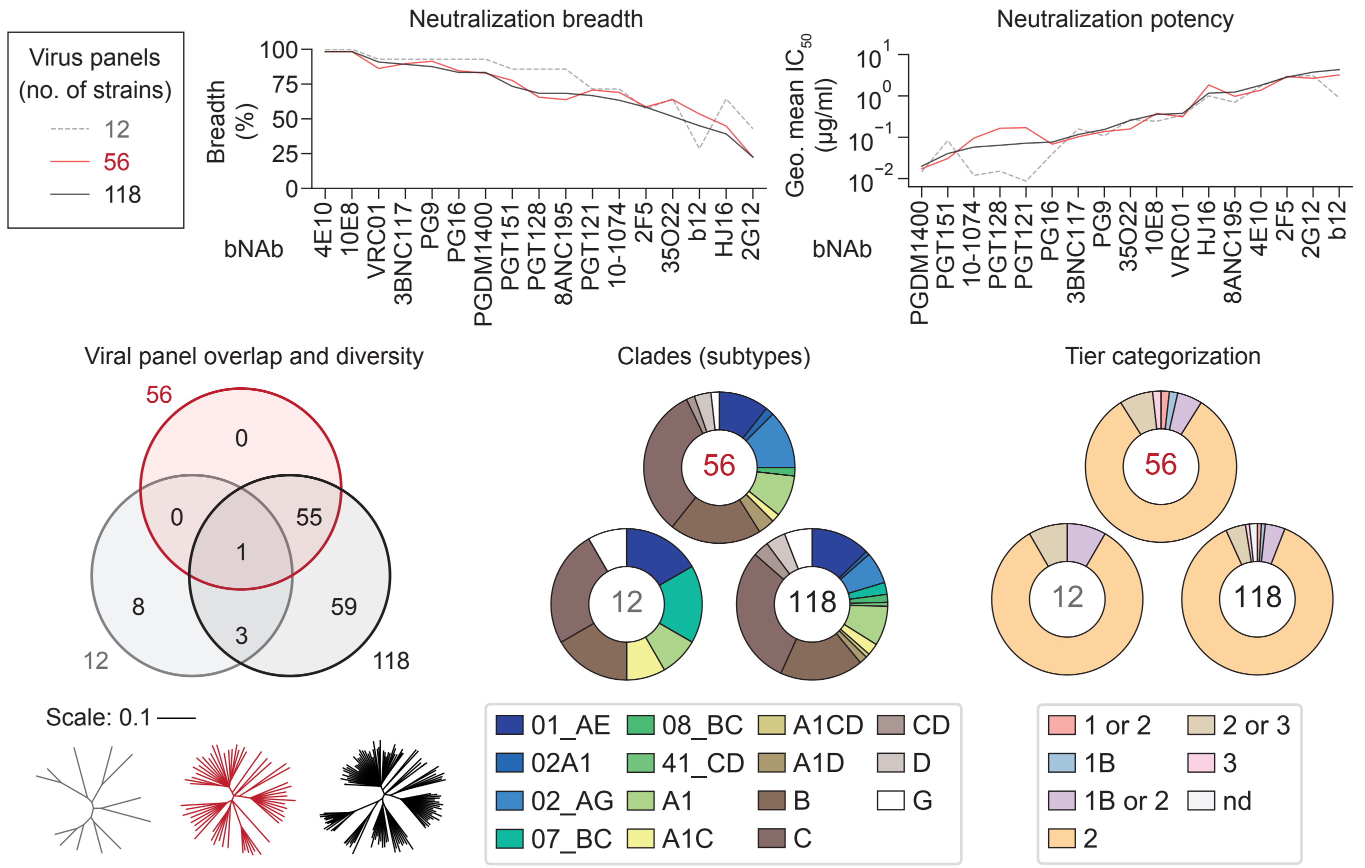

Supplementary Figure 4: Influence of viral panel composition on the determination of neutralization breadth and potency. Neutralization breadth and potency (geometric mean IC_{50} of neutralized strains) for 17 bNAbs based on three different viral panels: the 12 strain "global" panel (deCamp et al., 2014), the 118-strain multi-clade panel (Seaman et al., 2010), and a 56-strain subset panel, which was selected for the analyses in this work. The data for neutralization breadth and potency (upper part) is sorted by the values from the 118-strain panel. The lower part shows a comparison of the three panels in terms of overlap, phylogenetic trees, as well as clade composition and Tier categorization (lower part). Source data are provided as a Source Data file.

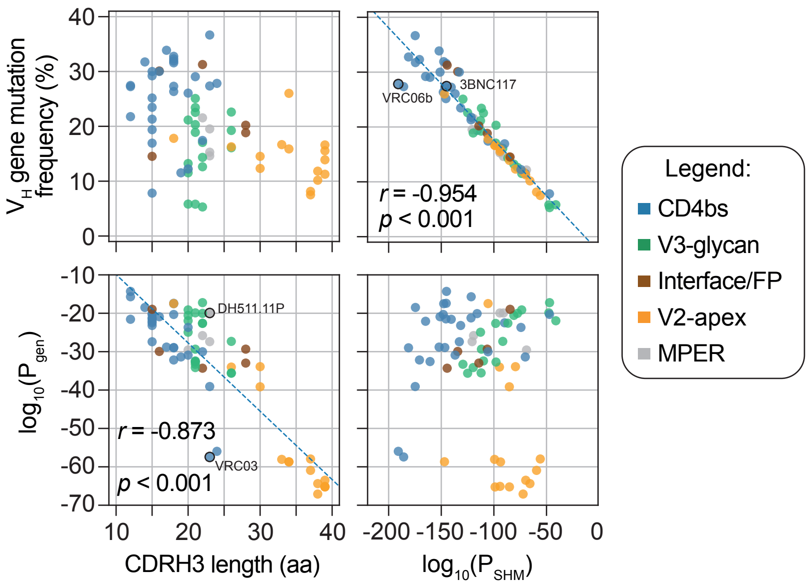

Supplementary Figure 5: Correlations of 70 bNAb heavy chain sequence features and probability values. Correlation coefficients (r) and two-sided p values were determined by linear regression (dashed line). Individual dots represent n=70 bNAb heavy chain sequences, colored by binding sites. Labeled antibodies are highlighted by black outlines. aa: amino acids. See Supplementary Data 4 and 5 for source data.

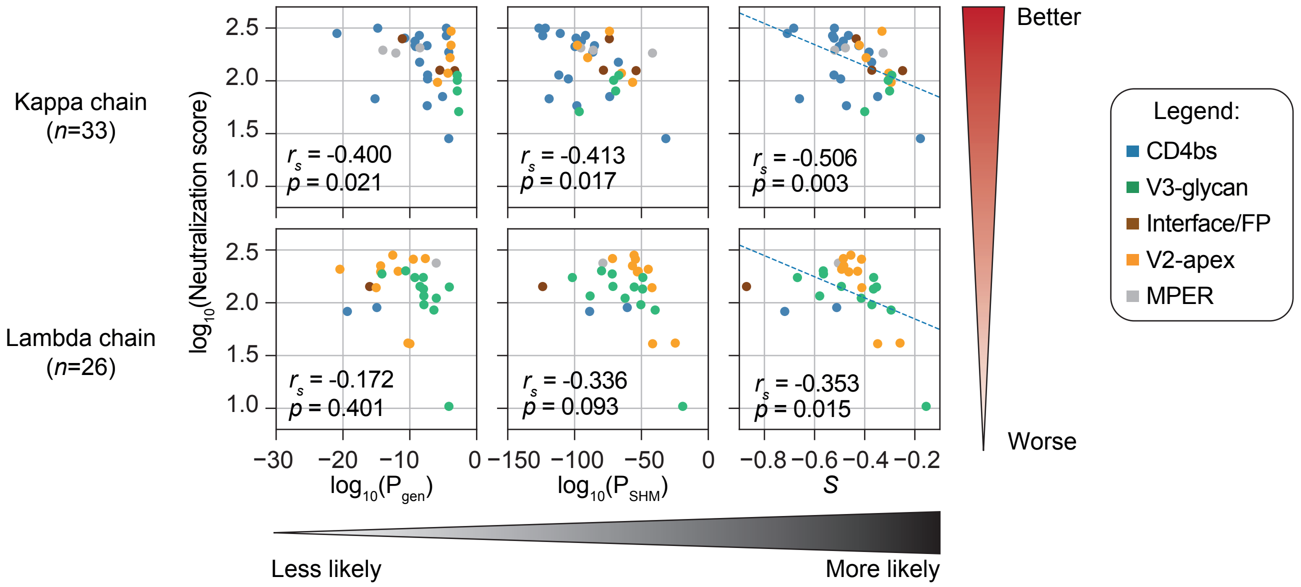

Supplementary Figure 6: Light chain probabilities of broadly neutralizing antibodies. Correlation plots of bNAb neutralization scores against light chain P_{gen}, P_{SHM} and probability scores $S = c_1\log_{10}(P_{gen}) + c_2\log_{10}(P_{SHM})$, separated by light chain isotype (*n*=33 kappa chains, *n*=26 lambda chains). Probability scores were derived by a linear regression (dashed line) with $c_1 = 1.404\times10^{-02}$ and $c_2 = 3.752\times10^{-03}$ for kappa and $c_1 = 1.202\times10^{-02}$ and $c_2 = 5.482\times10^{-03}$ for lambda chains. Spearman correlation coefficients r_s and two-sided *p* values are given in the figure. Correlation coefficients and two-sided *p* values from linear regressions for *S* are $r = -0.474$ and $p = 0.005$ for kappa and $r = -0.471$ and $p = 0.015$ for lambda chains, respectively. See Supplementary Data 4 and 5 for source data.

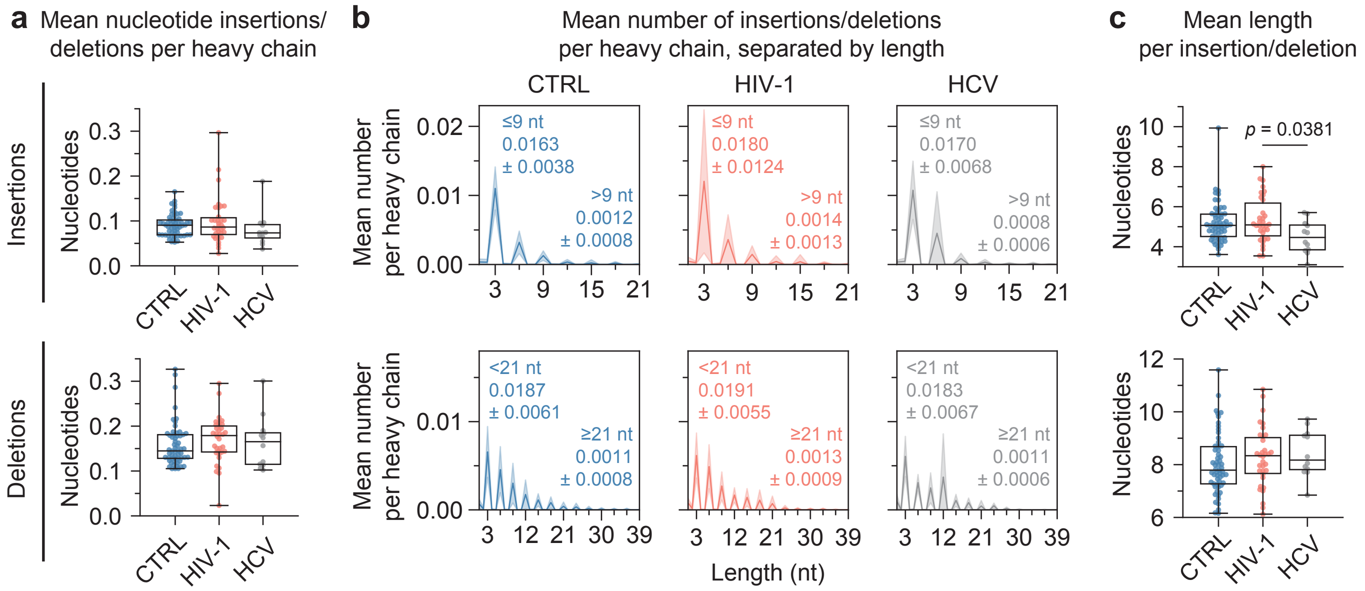

Supplementary Figure 7: Comparison of V gene segment insertions and deletions in IgG heavy chains between cohorts. (**a**) Mean numbers of nucleotide insertions/deletions per heavy chain V gene segment were determined for each individual (dots). Boxplots represent the 25% and 75% quantiles with medians as averages and min/max values as whiskers for uninfected (CTRL, n=57), HIV-1-infected (HIV-1, n=34), and HCV-infected (HCV, n=12) individuals. (**b**) Consecutive V gene segment nucleotide insertions and deletions were counted for each individual (CTRL, n=57; HIV-1, n=34; HCV, n=12) and the mean number of all distinct insertion/deletion lengths was determined per heavy chain. The lines depict the mean of each insertion/deletion length across all individuals. Shaded areas represent standard deviations. (**c**) The mean length of V gene segment insertions/deletions was determined for each individual (dots; CTRL, n=57; HIV-1, n=34; HCV, n=12) and summarized as in (a) for each cohort as boxplots. Differences in mean lengths were determined by one-way ANOVA and Tukey post hoc test. nt: nucleotide. Source data are provided as a Source Data file.

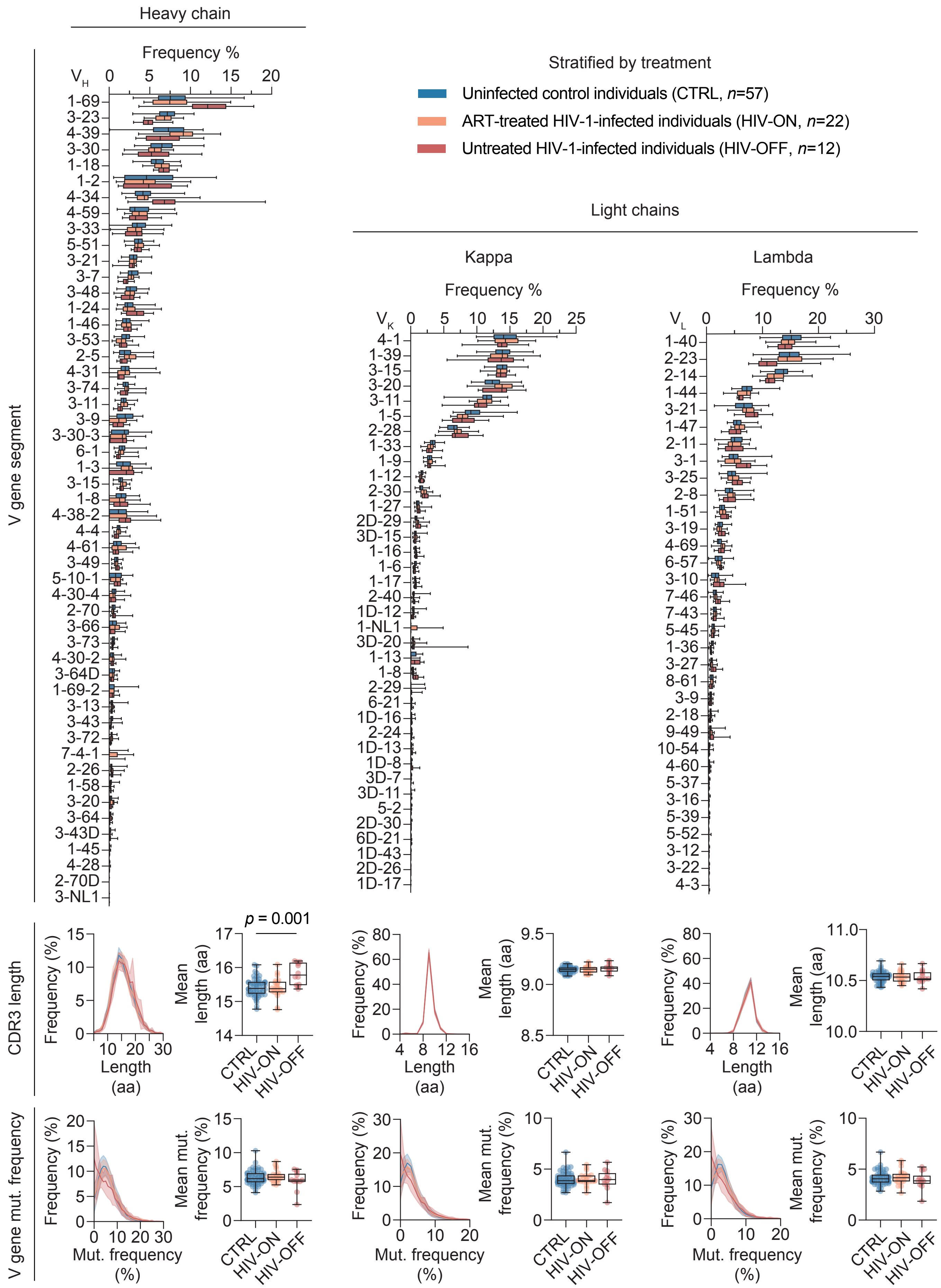

Supplementary Figure 8: IgG heavy chain and light chain repertoire characteristics, stratified by antiretroviral treatment. Heavy and light chain V gene segment usage, CDR3 length, and V gene mutation frequency distributions for uninfected (CTRL, *n*=57), as well as treated (HIV-ON, *n*=22) and untreated (HIV-OFF, *n*=12) HIV-1-infected individuals. Differences in mean CDR3 lengths were determined by one-way ANOVA and a two-sided Tukey post hoc test. Distributions show means as solid lines and standard deviation as shaded area. Box-plots show 25% and 75% percentiles with medians as average lines as well as the minimum and maximum values as whiskers. aa: amino acids, mut.: mutation. Source data are provided as a Source Data file.

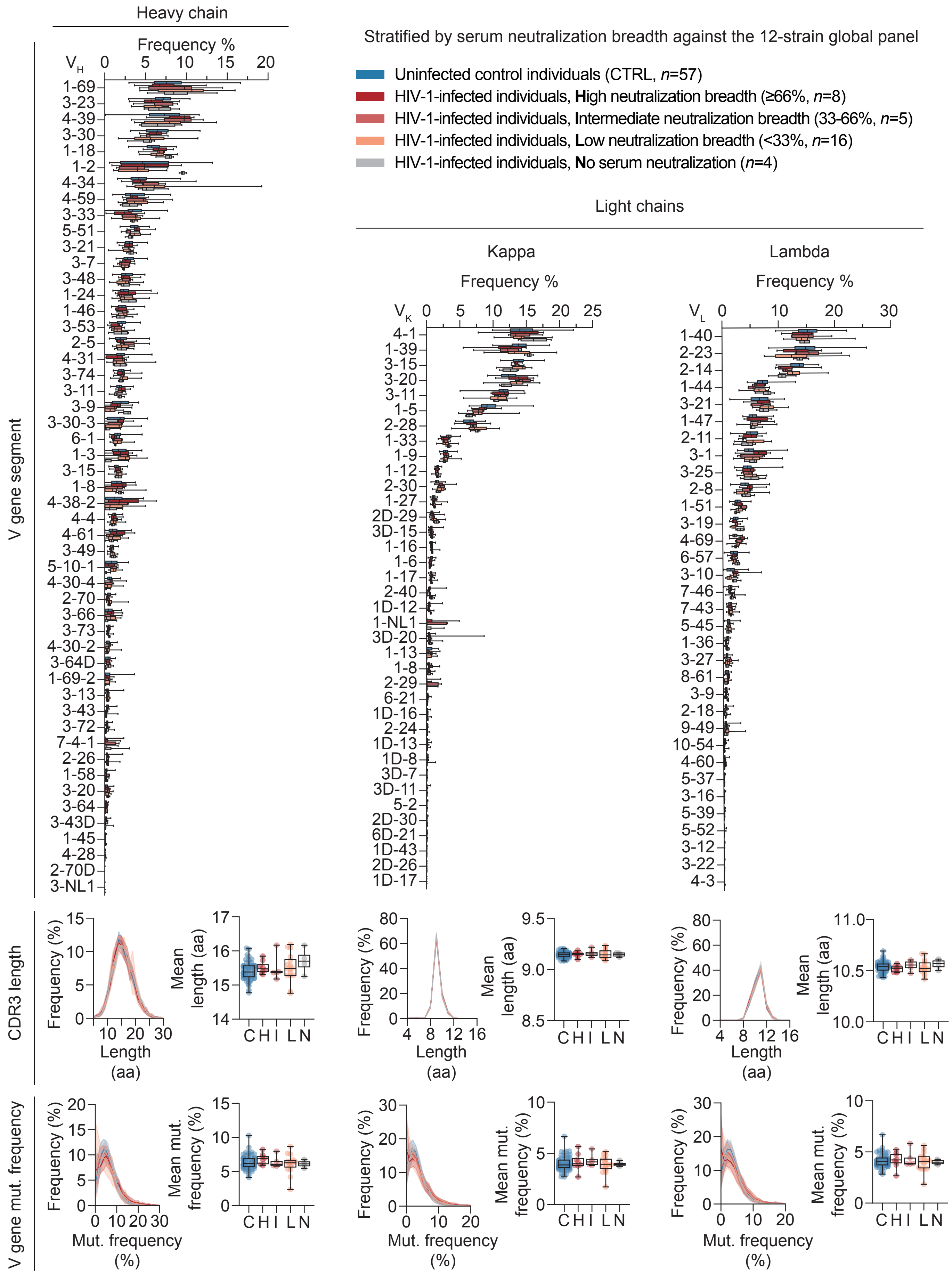

Supplementary Figure 9: IgG heavy and light chain repertoire characteristics, stratified by neutralization activity. Heavy and light chain V gene segment usage, CDR3 length, and V gene mutation frequency distributions for uninfected (CTRL, *n*=57), as well as HIV-1-infected individuals (*n*=34) who have been grouped by their serum neutralization breadth against the 12-strain global panel into high (H, *n*=8), intermediate (I, *n*=5), low (L, *n*=16), and no neutralization breadth (N, *n*=4). Distributions show means as solid lines and standard deviation as shaded area. Box-plots show 25% and 75% percentiles with medians as average lines as well as the minimum and maximum values as whiskers. aa: amino acids, mut.: mutation. Source data are provided as a Source Data file.
