# Supplementary material for: Probabilities of developing HIV-1 bNAb sequence features in uninfected and chronically infected individuals
Source: Nat Commun. 2023 Nov 6;14:7137. doi: 10.1038/s41467-023-42906-y (PMC10628170; doi:10.1038/s41467-023-42906-y)
Supplement: Supplementary file 3 — Description of Additional Supplementary Files [file 41467_2023_42906_MOESM3_ESM.pdf]

Description of Additional Supplementary files

"Probabilities of developing HIV-1 bNAb sequence features in uninfected and chronically infected individuals"

File name: Supplementary Data 1
Description: Control experiment processing and sequencing statistics.

File name: Supplementary Data 2
Description: Uninfected cohort demographics and sequencing statistics.

File name: Supplementary Data 3
Description: Viral panel overview.

File name: Supplementary Data 4
Description: Features of HIV-1 broadly neutralizing antibodies.

File name: Supplementary Data 5
Description: Heavy and light chain P_{gen}, P_{SHM}, and probability scores.

File name: Supplementary Data 6
Description: HIV-1 cohort demographics and sequencing statistics.

File name: Supplementary Data 7
Description: HCV cohort demographics and sequencing statistics.

File name: Supplementary Data 8
Description: HIV-1 cohort poly IgG neutralization on the 12-strain global panel.
